# Discovery of Novel Inhibitors of Indoleamine 2,3-Dioxygenase 1 Through Structure-Based Virtual Screening

**DOI:** 10.3389/fphar.2018.00277

**Published:** 2018-03-29

**Authors:** Guoqing Zhang, Jing Xing, Yulan Wang, Lihao Wang, Yan Ye, Dong Lu, Jihui Zhao, Xiaomin Luo, Mingyue Zheng, Shiying Yan

**Affiliations:** ^1^School of the Physical Sciences, Qingdao University, Qingdao, China; ^2^State Key Laboratory of Drug Research, Drug Discovery and Design Center, Shanghai Institute of Materia Medica, Chinese Academy of Sciences, Shanghai, China; ^3^Department of Pharmacy, University of Chinese Academy of Sciences, Beijing, China; ^4^School of Science, East China University of Science and Technology, Shanghai, China

**Keywords:** indoleamine 2, 3-dioxygense, IDO1 inhibitor, virtual screening, molecular docking, hit expansion

## Abstract

Indoleamine 2,3-dioxygenase 1 (IDO1) is an intracellular monomeric heme-containing enzyme that catalyzes the first and the rate limiting step in catabolism of tryptophan via the kynurenine (KYN) pathway, which plays a significant role in the proliferation and differentiation of T cells. IDO1 has been proven to be an attractive target for anticancer therapy and chronic viral infections. In the present study, a class of IDO1 inhibitors with novel scaffolds were identified by virtual screening and biochemical validation, in which the compound DC-I028 shows moderate IDO1 inhibitory activity with an IC_50_ of 21.61 μM on enzymatic level and 89.11 μM on HeLa cell. In the following hit expansion stage, DC-I02806, an analog of DC-I028, showed better inhibitory activity with IC_50_ about 18 μM on both enzymatic level and cellular level. The structure–activity relationship (SAR) of DC-I028 and its analogs was then discussed based on the molecular docking result. The novel IDO1 inhibitors of DC-I028 and its analogs may provide useful clues for IDO1 inhibitor development.

## Introduction

Indoleamine 2,3-dioxygenase 1 (IDO1) degrades the essential amino acid L-tryptophan (L-Trp) into kynurenine (KYN), which is known as the initial and the rate limiting step of the KYN pathway (Takikawa et al., 1986). The catabolism of tryptophan produces a series of biologically active metabolites in various physiological and pathological processes, including neurotransmitters serotonin, KYN, 3-hydroxyanthranilic (3-HAA), quinolinic acid (QUIN), N-methyl-D-aspartate (NMDA) receptor antagonist, kynurenic acid, and nicotinamide adenine dinucleotide (NAD) (Takikawa et al., 1986; Bessede et al., 2014). The degradation of tryptophan and accumulation of tryptophan metabolites would inhibit the proliferation and differentiation of T cells (John et al., 2010; Bessede et al., 2014). Three enzymes, namely IDO1, indoleamine 2,3-dioxygenase 2 (IDO2), and tryptophan 2,3-dioxygenase (TDO) are found controlling this step, but only IDO1 is proved to be sensitive in many cancer types. This is because TDO expression has been implicated in cancer states, and IDO2 is inactive in some common polymorphisms (John et al., 2010). Thus, IDO1 acts as a direct guard against T-cell attacking and overexpressed in many human cancer types (Hwu et al., 2000; Austin and Rendina, 2015), including breast cancer (Mansfield et al., 2009), prostate cancer (Kallberg et al., 2010), lung cancer (Astigiano et al., 2005), and colon cancer. Moreover, IDO1 is also expressed in antigen presenting cells, which enhances peripheral tolerance to tumor associated antigens (TAAs) in tumor draining lymph nodes (Astigiano et al., 2005; Ferdinande et al., 2012). By protecting the malignant cells expressing TAAs from the recognition and attack of the immune system, IDO1 promotes the survival, invasion, growth, and metastasis of these cells (Curti et al., 2009; Ferdinande et al., 2012). *In vivo* studies using animal models of human cancers treated with IDO1 inhibitors demonstrated that IDO1 may be a potential therapeutic target for the cancer immunotherapy (Curti et al., 2009).

**Figure 1:**
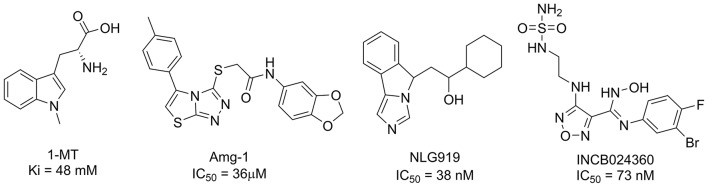
Several published IDO1 inhibitors.

To date, several classes of IDO1 inhibitors showed potent efficacy when used alone or in combination with other therapeutics in clinical trials (Cady and Sono, 1991; Liu et al., 2010; Smith et al., 2012a; Mautino et al., 2013; Peng et al., 2016; Brochez et al., 2017). The most frequently used inhibitor of IDO1, 1-methyl-D-tryptophan (D-1MT) developed by NewLink Genetics, is currently undergoing different clinical trials (Brochez et al., 2017). Another competitive inhibitor Amg-1 reported by Meininger et al. showed good inhibitory activity and selectivity (Meininger et al., 2011). In addition, Mautino et al. identified an imidazole derivative NLG919 (Mautino et al., 2013) as an IDO1 inhibitor, which was later licensed to Genentech. NLG919 is undergoing a Phase I clinical trial for the treatment of recurrent advanced solid tumors. Another small molecule IDO1 inhibitor INCB024360 (also named as epacadostat) developed by Incyte, Inc., shows potent inhibitory activity against IDO1 (Yue et al., 2009). *In vivo* study revealed that INCB024360 was efficacious as a single agent or in combination with immunotherapy and cytotoxic agents in cancer models. Currently, INCB024360 is evaluated in several clinical trials for the treatment of advanced or metastatic cancers (Kristeleit et al., 2017). Figure 1 summarizes representative IDO1 inhibitors with different chemotypes. According to the report on Thomson Reuters Integrity, 11 published IDO-1 inhibitors have advanced into clinical trial stage. However, five of them are tryptophan or purine derivatives, including Indole-3-carbinol, Diindolylmethane, Indoximod, EOS-200271, and Dezaguanine. Three of them are from natural products (Epigallocatechin gallate, β-Lapachone, and Aminogeldanamycin). Given their limited structural diversity and optimization prospects, it is therefore of high interest to discover IDO1 inhibitors with novel scaffolds.

Compared with traditional bio-assay hit screening methods, virtual screening exhibited higher throughput and lower cost. With the rapid development of computational methods, virtual screening has been successfully used in many hit compound discovery projects (Ou-Yang et al., 2012; Zheng et al., 2013; Coluccia et al., 2016). For example, Smith et al. (2012b) developed an *in silico* screening protocol utilizing multiple pharmacophores in conjunction with docking techniques to screen the Maybridge compound library (59,652 molecules) and resulted 20 candidates, among which 4 hits were identified by IDO-1 enzymatic assay. In this study, from structure-based virtual screening, we successfully identified a novel IDO1 inhibitor DC-I028 (IC_50_ = 21.61 μM). Putative binding modes of DC-I028 in the heme binding site of IDO1 were analyzed before hit expansion. Among the resulted analog, DC-I02806 showed better inhibitory activity. Furthermore, the cellular activity assay showed DC-I028 and its analogs could inhibit IDO1 in HeLa cells with similar activity of that on molecular level (Graphical Abstract).

**Graphical Abstract:**
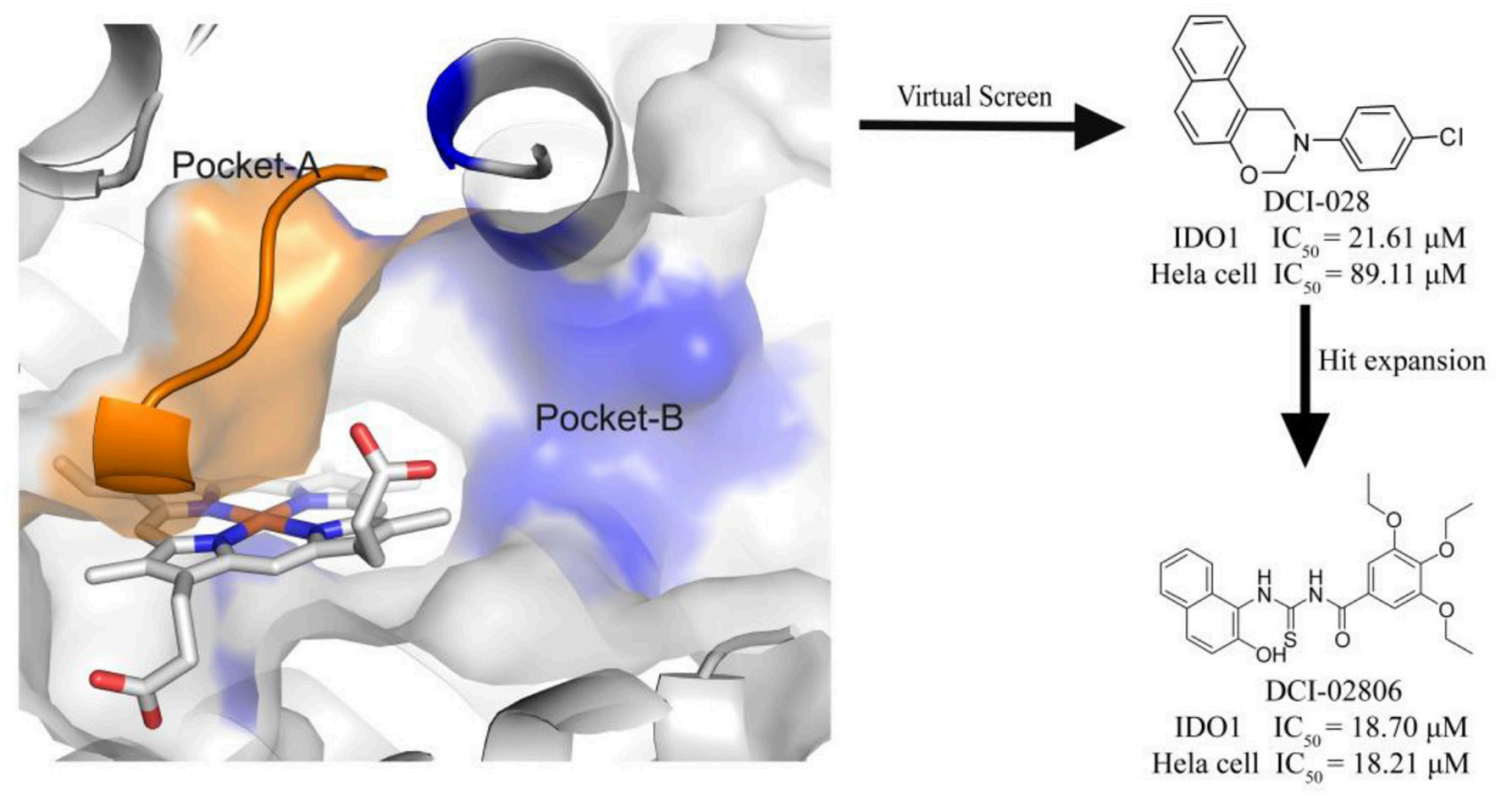
A novel IDO1 inhibitor, DCI-028, was discovered by a virtual screening model based on the 3D structure of the target. Further hit expansion work brought a more potent molecule, DCI-02806.

## Methods

### Ligand preparation

Pan-Assay Interference compounds (PAINS) (Baell and Holloway, 2010; Capuzzi et al., 2017) and non-drug-like compounds (Lipinski et al., 2001) were removed from the compound library with Pipeline Pilot (version 7.5, Accelrys Software Inc.: San Diego, CA). Then LigPrep (version 2.4, Schrödinger, LLC, New York, NY, 2010) was used to generate stereoisomers and tautomers, and the protonation states of ligands at pH 7.0 ± 2.0 were generated with Epik (Shelley et al., 2007). For other parameters, the default values were assigned.

### Protein preparation

Currently, there are 10 available X-ray crystal structures of IDO1 complexed with different inhibitors in the Protein Data Bank, and their PDB codes are 2D0T, 2D0U, 4PK5, 4PK6, 4U72, 4U74, 5EK2, 5EK3, 5EK4, and 5ETW, respectively. These structures were prepared with the Protein Preparation Wizard Workflow provided in the Maestro module of Schrödingersoftware (Schrödinger, LLC: New York, NY, 2015). The protein structure was firstly fixed by verifying proper assignment of bonds, adding hydrogens, creating zero-order bong to metals, creating disulfide bonds, deleting water molecules beyond 5 Å from the het group, removing waters with less than 3 H-bonds to non-waters, and a restrained minimization that allows only hydrogen atoms to be freely minimized. Subsequently, the receptor grids of these complexes were generated with Glide module of Schrödinger software, and the grid boxes were defined as a 20 × 20 × 20 Å space region centered at the original ligand of the complex structures. In addition, one metal-coordination was introduced into heme iron during grid box generation. For the other parameters, the default values were assigned.

### Docking model selection and structure-based molecular docking targeting the IDO1 pocket

Published IDO1 inhibitors were selected as positive compounds, and their decoys generated by DUD•E (Mysinger et al., 2012) were regarded as negative compounds. Then, all the prepared inhibitors and decoys were docked into the binding site of IDO1 with Glide (version 5.6, Schrödinger, LLC, New York, NY, 2010) in standard precision (SP) mode. In order to select a proper crystal structure of IDO1 for virtual screening, we calculated the enrichment factor (EF) (Halgren et al., 2004) for every crystal structure with an Enrichment Calculator of Maestro. EF is used to evaluate the ability of a virtual screening model to identify actives from inactives, which was defined as Equation (1):

(1) $$\begin{array}{r} EF{=}^{\frac{Hits_{set}}{n}}{/}_{\frac{Hits_{all}}{N}} \end{array}$$

where ***Hits***_***set***_ is the number of actives in the selected subset ***n*** of the ranked database and ***Hits***_***all***_ is the total number of actives in the database of ***N*** compounds. Then the model with the highest EF values was employed for docking screening. The top-ranked compounds were clustered based on 2D-structural similarity (Tanimoto coefficient of the ECFP4 fingerprint) in Pipeline Pilot (version 7.5, Accelrys Software Inc.: San Diego, CA) before visual inspection.

### Hit expansion

Structural similarity searching and substructure filtering were performed in Pipeline Pilot (version 7.5, Accelrys Software Inc.: San Diego, CA) to explore the chemical space of the hit compound. Similarity searching was conducted by ECFP4 Tanimoto coefficient calculation with the hit compound taken as the query and the similarity threshold set to 0.5. The putative scaffolds of the hit compound were employed as the substructure query to filter the compound library. Finally, to ensure the rationality of the analog, the results of similarity searching and substructure filtering were checked manually.

### Enzymatic IDO1 assays

The standard assay mixture contained modified potassium phosphate buffer at pH 6.5, 100 μM L-tryptophan (Sigma, Cat. No. 93659-10G), ascorbate (Signma, Cat. No. 11140-250G), methylene blue (Sigma, Cat. No. M9140-100G), and catalase (Sigma, Cat. No. C9322-5G). The human IDO1 protein (ChemPartner, Lot. No. 20160706), was pre-incubated with a range of compound concentrations or a single dose for 15 min at room temperature, and then added substrate mix solution. For 100% inhibition control (Min), 1 X assay buffer was used instead of IDO1 enzyme solution. And for no inhibition control (Max), DMSO (Sigma, Cat. No. 3635) was used instead of compound DMSO solution. Then the assay plate was read at OD321 kinetically in Spectramax. The inhibition percentage in the presence of the compound was calculated according to the equation, Percent inhibition = (Max – Signal)/(Max – Min) ^*^ 100%. Fit the data in GrphaPad Prism V5.0 software to obtain IC_50_-values using Equation (2):

(2) $$\begin{array}{r} Y=Bottom+\frac{Top-Bottom}{1+10^{\left(\log\text{I}{\text{C}}_{50}-X\right)\times HillSlope}} \end{array}$$

where *Y* stands for inhibition percentage and *X* stands for compound concentration. All determinations were carried out in duplicate.

### Hella cellular IDO1 assays

We chose HeLa cell line (ATCC, Cat. No. CCL-2, 4965442) to detect whether our compounds could inhibit IDO activity at cellular level by optical density (OD) assay. The assay was performed in 96-well flat bottom plate (CORNING, Cat. No. 3903) seeded with 5,000 cells in a final volume of 100 μL. Each well 80 μL HeLa cells were incubated overnight at 37°C in EMEM (Invitrogen, Cat. No. 11095098) supplemented with 10% FBS (Invitrogen, Cat. No. 10099-141, Lot. No. 8153379). Then compounds that have been serial diluted were added in corresponding well and 10 μL Recombinant Human IFN-γF (Peprotech, Cat. No. 300-02) was added in each well except wells for Min (Medium containing 0.5% DMSO with cells). The mixture of cells were incubated at 37°C, 5% CO_2_ for 48 h. Then transferred 90 μL of supernatant to 96-well plate (CORNING, Cat. No. 3599) and store at −20°C fridge for colormetric analysis. The next day, 10 μL of 6.1 N trichloroacetic acid (Sigma, Cat. No. T0699) were added in each well after transferred 80 μL supernatant to 96-well plate (CORNING, Cat. No. 3894). Then the mixture was incubated at 50°C for 30 min. Sediments were removed by centrifuged for 10 min at 2,500 rpm. Seventy microliters of 70 μL of 2% (w/v) p-dimethylaminobenzaldehyde (Sigma-Aldrich, Cat. No. 15647-7) was added after 70 μL of the supernatant were transferred to assay plate (CORNING, Cat. No. 3635). Finally, OD-value was measured at 480 nm using an EnSpire (PE).

## Results and discussion

### Molecular docking targeting the IDO1 pocket

In order to choose an appropriate crystal structure for virtual screening of IDO1 inhibitors, we compared their capability for enriching known active compounds in the top scored compounds. Ninety previously published IDO1 inhibitors were collected from published literatures (Table S1) and selected to generate 6,000 decoy compounds. For each available IDO1 crystal structure, a separate molecular docking model was constructed, and the results of the enrichment calculation are shown in Table 1. The crystal structure of PDB ID 4U74 showed the best results with the area under the receiver operating characteristic curve (AUC-ROC) of 0.71, Robust Initial Enhancement (RIE) of 4.59 and EF (top 20%) of 3.2. Therefore, 4U74 (PDB ID) were selected for subsequent virtual screening.

**Table 1:** Results of Enrichment Calculator.

**PDB ID**	**AUC-ROC**	RIE^*a^	EF^*b^ 1%	**EF 5%**	**EF 20%**
2D0T	0.72	4.31	10	5.3	2.8
4PK5	0.67	1.82	1.1	2.7	2.1
4PK6	0.64	1.17	1.1	1.6	1.5
4U72	0.67	3.97	11.1	5.3	2.6
4U74	0.71	4.59	12.2	6.4	3.2
5EK2	0.68	3.81	11.1	4	2.1
5EK3	0.56	2.95	6.7	2.9	1.7
5EK4	0.57	2.73	8.9	2.2	1.6
5ETW	0.65	2.79	10	2.4	1.9

*a *RIE, robust initial enhancement (Truchon and Bayly, 2007)*.

*b *EF, enrichment factors*.

### Structure-based virtual screening

**Figure 2:**
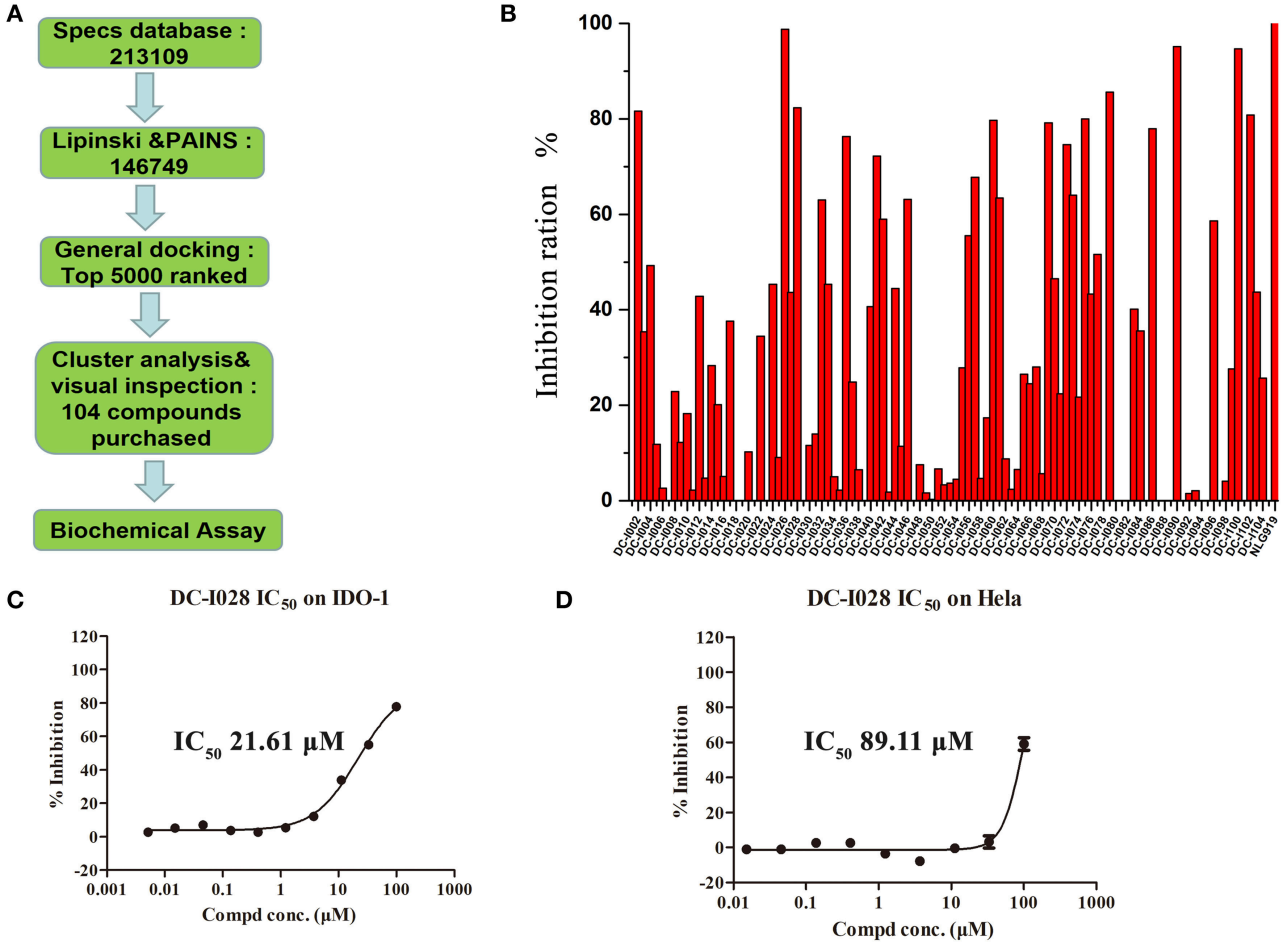
Virtual screening procedures and activity assays for IDO1 *in vitro*. **(A)** Flowchart of the integrated virtual screening for IDO1 inhibitors. Numeral indicates the number of molecules in each stage. **(B)** Inhibitory activity of the 104 candidate molecules at 50 μM. The red columnar bars represent the inhibitory activity of compounds against IDO1. NLG919 was the reference compound. **(C)** The inhibitory activity of DC-I028 against IDO1 at enzymatic level. **(D)** The IDO1 inhibitory activity of DC-I028 in Hela cells.

The overall workflow of structure-based virtual screening was shown in Figure 2A. The Specs database (http://www.specs.net) containing more than 210,000 molecules was used as ligand database for virtual screening. The ligand database was firstly filtered using Lipinski's rule of five, and then the compounds containing substructures of PAINS were removed. The remaining 146,749 molecules were prepared to dock into the catalytic site of IDO1 (PDB ID: 4U74). The top 5,000 compounds ranked by Glide docking score were selected and clustered into 200 groups. The compound of each cluster was selected manually with the following principles: (1) Lower molecular weight; (2) Forming metal-ligand interaction with the heme iron; (3) Forming hydrogen bond with chain Ala260-Gly265. Finally, 104 compounds were selected and purchased from Specs for further biological assay evaluation.

### IDO1 inhibitory activity

The Absorbance 321 assay on IDO1 was used to determine the inhibitory activity of the selected 104 compounds (Table S2). Among these compounds, 23 compounds showed inhibition ability against IDO1 ≥ 50% at 50 μM (Figure 2B). Considering the inhibition ratio and background signal of the compounds (Table S2), we chose 6 candidate compounds with high inhibitory activity or low background signal from the above 23 compounds and tested their IDO1 inhibitory activity at 20 μM. As shown in Table 2, compound DC-I028 showed 82% inhibition ratio to IDO1 at 50 μM and 58% inhibition ratio at 20 μM. The IDO1 enzymatic IC_50_ of DC-I028 was 21.61 μM (Figure 2C). In Hela cells, it achieved IDO1 IC_50_ of 89.11 μM (Figure 2D). Therefore, DC-I028 was selected for further study.

**Table 2:** Inhibition ratio data of six candidate hits of virtual screening, as measured by the Absorbance 321 assay on IDO1.

CPD No.^*^	**Inhibition ratio (%) @ 50 μM**	**Inhibition ratio (%) @ 20 μM**	**Enzymatic IC_50_ (μM)**	**Hela IC_50_ (μM)**
DC-I028	81.62	57.62	21.61	89.11
DC-I032	63.02	25.26	–	–
DC-I046	63.18	28.10	–	–
DC-I079	85.60	36.42	–	–
DC-I090	95.14	41.43	–	–
DC-I102	80.83	15.17	–	–
NLG919	100.00	100.00	–	–

* *CPD No., compound number*.

### Binding mode prediction

**Figure 3:**
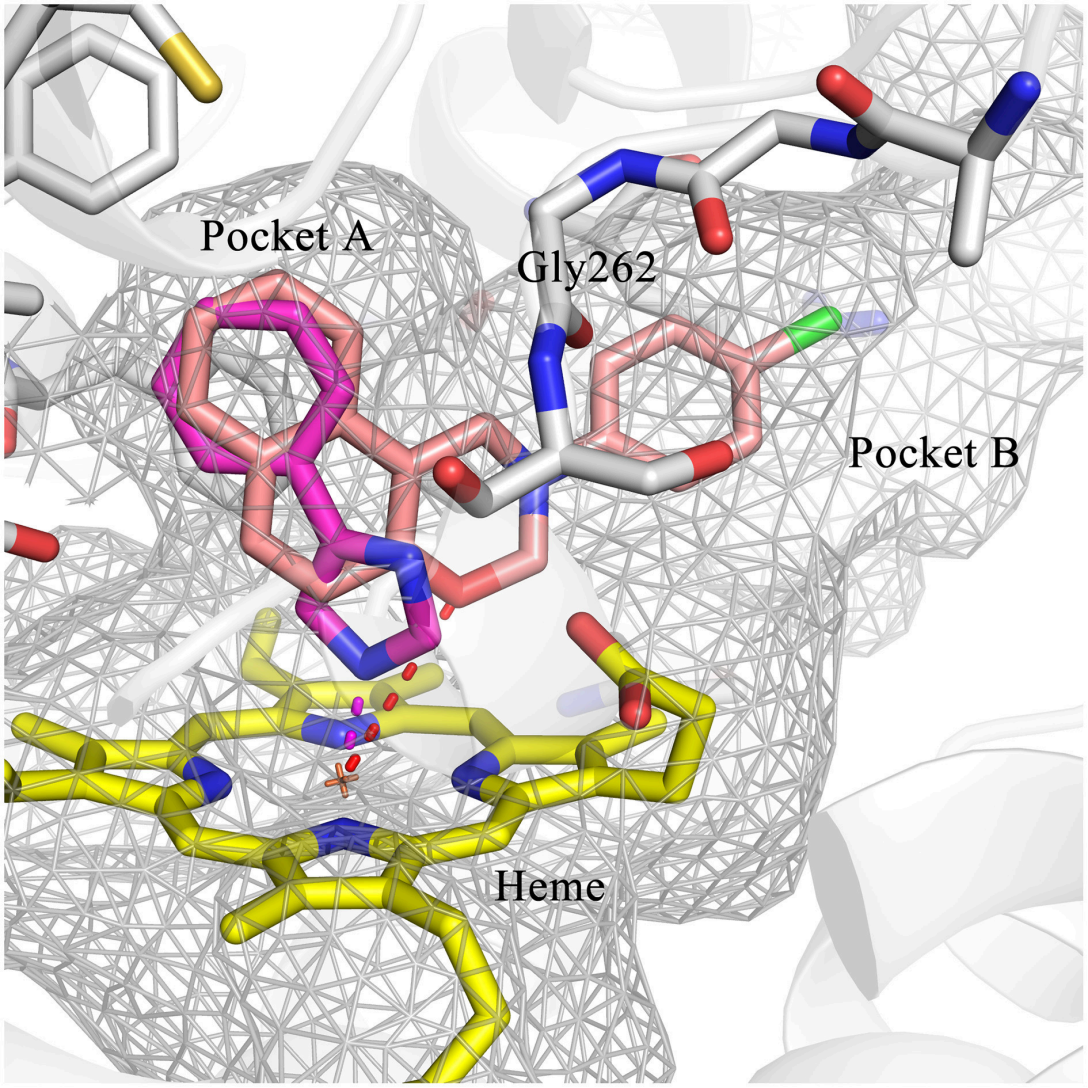
Comparison of the binding modes of 4-PI (magenta) and DC-I028 (orange) in the active site of IDO1 (PDB ID: 4U74). Interactions with metal were shown as dashed lines. Key residues for ligand binding are shown as sticks. The surface of the pocket was shown as wireframes. The figure depicting the binding modes was generated with PyMOL (Schrodinger, 2015).

To explore the molecular basis of the inhibitory activity, molecular docking studies were performed. The predicted binding mode of DC-I028 in the heme binding site of IDO1 was compared with the X-ray co-crystal structure of the IDO1/4-PI [(4-phenylimidazole, 4-PI), which was firstly identified as a weak noncompetitive IDO1 inhibitor (IC_50_ = 143 μM); PDB ID 4U74] (Kumar et al., 2008). As shown in Figure 3, DC-I028 displays a similar binding mode to 4-PI, and the imidazole oxygen atom of DC-I028 formed interaction with the heme iron. In the binding pocket A, the naphthalene ring of DC-I028 is aligned well with the phenol group of 4-PI, and both of them form “T-shaped” π-π interactions with Tyr-126. The major difference between the binding modes of these two compounds is at the binding pocket B, where the 4-Cl-phenyl group of DC-I028 forms extra hydrophobic interactions. The interactions with the pocket B may explain the better inhibition of IDO1 by DC-I028, as compared with 4-PI.

### Hit expansion and SAR analysis

**Figure 4:**
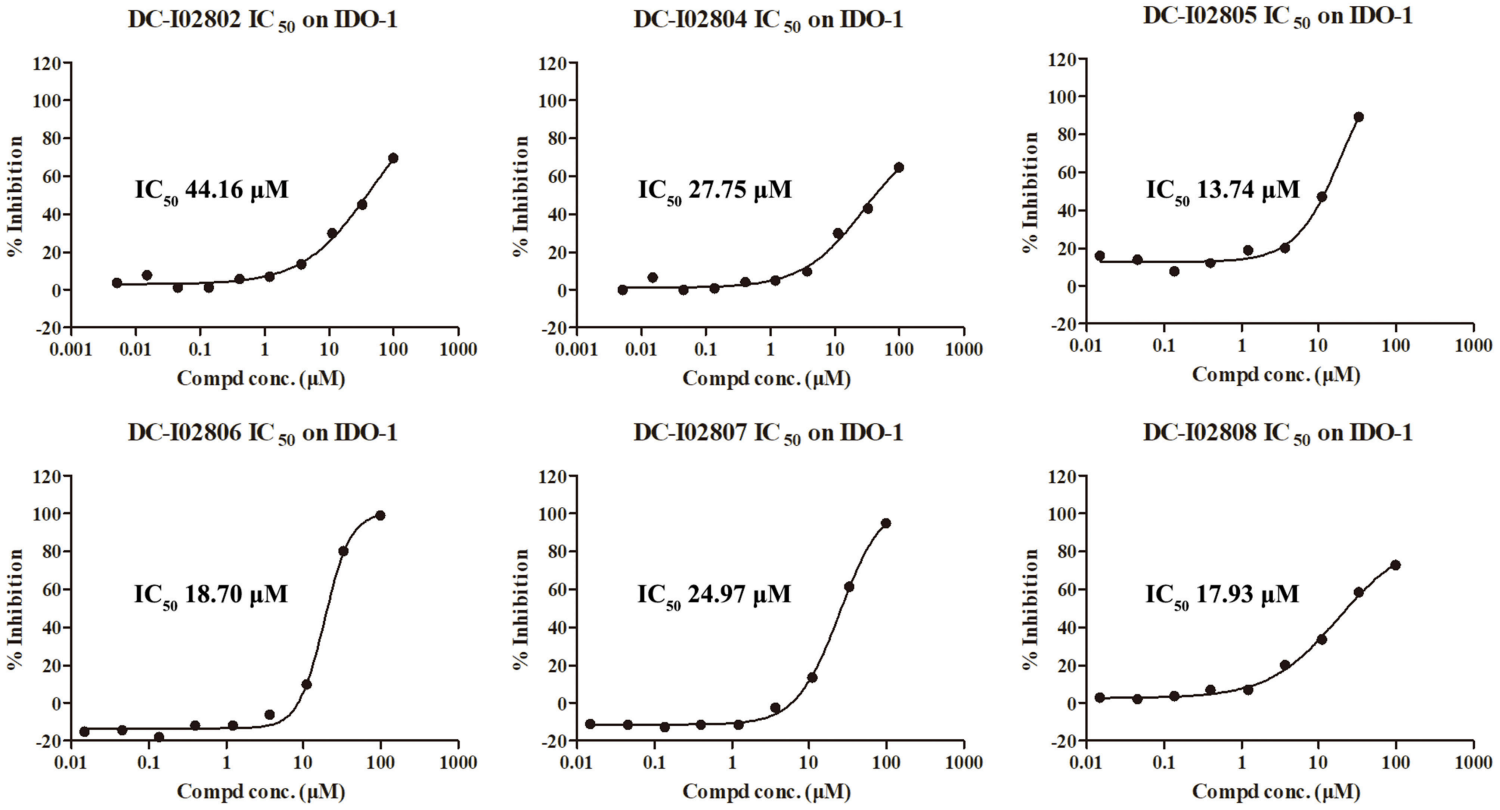
IDO-1 enzymatic inhibition of analog of DC-I028 and their dose-response behaviors.

**Figure 5:**
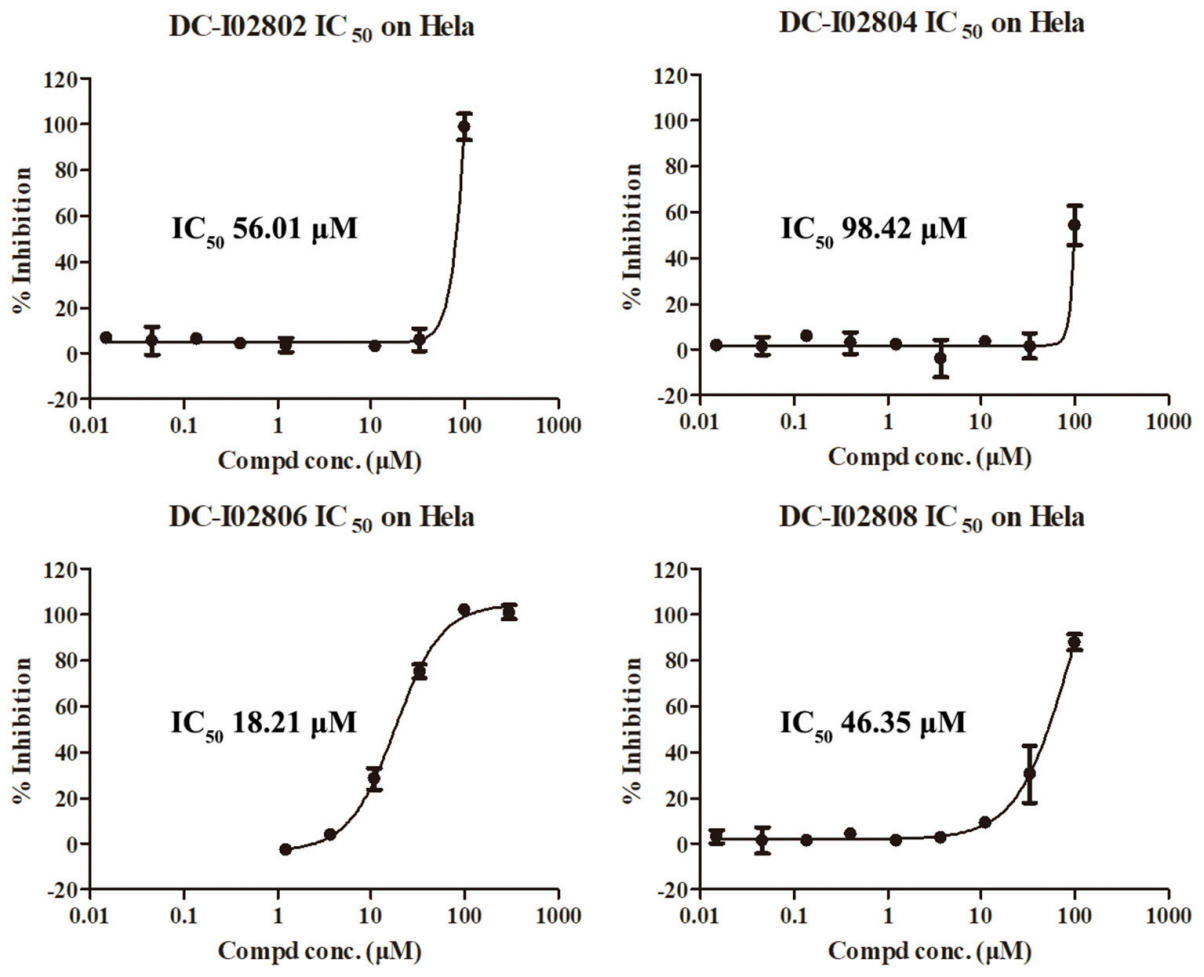
IDO-1 inhibition in Hela cells of analog of DC-I028 and their dose-response behaviors.

**Figure 6:**
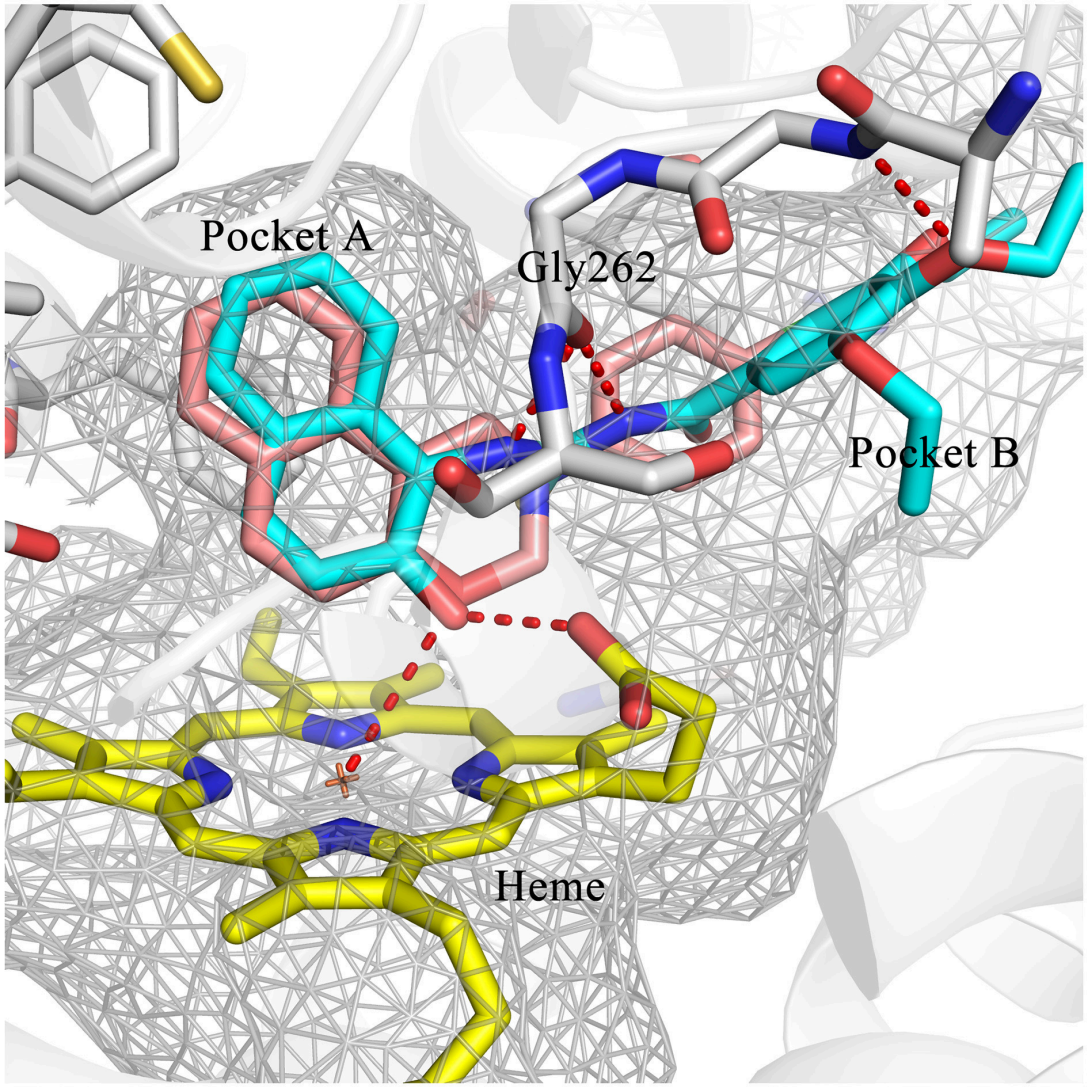
Comparison of the binding modes of DC-I028 (orange) and DC-I02806 (cyan) in the active site of IDO1 (PDB ID: 4U74). Metal and hydrogen bonding interactions were shown as red dashed lines. Key residues for ligand binding are shown as sticks. The surface of the pocket was shown as wireframes. The figure depicting the binding modes was generated with PyMOL (Schrodinger, 2015).

Hit expansion was conducted to explore the SAR of compound DC-I028. Thirteen analogs of DC-I028 were found and determined inhibitory activity (Table 3). Most of them exhibited dose-dependency at enzymatic level (Figure 4) and cellular level (Figure 5). As shown in Table 3, five compounds displayed inhibition against IDO1 of more than 40% at 20 μM and some IC_50_-values against IDO1 were determined. The results suggested that the analogs of DC-I028 also exhibited inhibitory activity against IDO1, which further indicated DC-I028 could be characterized as a structurally novel IDO1 inhibitor. We further analyzed the SARs of DC-I028 and its analogs to find clues for future structural optimization. As shown in Table 3, their structures could be divided into three types. In scaffold (I), the binding affinity was decreased when the R_1_ group was replaced by para-acetylphenyl (DC-I02801), but retained by R_1_ groups with ortho- and meta- substitutions on the phenyl (DC-I02802 and DC-I02804). For the inactive DC-I02803, the moiety of naphthalene ring was added a substitution of acetamido group (R_2_ group), which might clash with the pocket according to the predicted binding pose of DC-I028 (Figure 3). In scaffold (II), the oxazine moiety of DC-I028 was changed as opening substituents of R_1_ and substituted thiourea group (R_2_). In the analogs of scaffolding (II), higher binding affinities were observed for hydroxyl substitution at R_1_ (DC-I02805 to DC-I02808), compared with hydrogen (DC-I02809 and DC-I02810). According to the binding mode predicted by docking (Figure 6), the hydroxyl R_1_ group of the most potent compound DC-I02806 might form hydrogen bonding with Heme, also R_2_ might form two hydrogen bonds with Gly262. However, no hydrogen bond between DC-I028 and Gly262 was observed according to its docked pose, which could explain the activity increase of DC-I02806. Meanwhile, compared with the analogs of scaffolding (II), the analogs of scaffolding (III) DC-I02811, DC-I02812, and DC-I02813 lacked the substituted thiourea group, which could act as a hydrogen bond donor to Gly262 (Figure 6), and their inhibitory activity decreased dramatically. From this hit expansion research, we discovered a more potent IDO1 inhibitor, DC-I02806, which exhibited IDO1 inhibition with IC_50_ about 18 μM at both molecular and cellular levels.

**Table 3:** Inhibition of IDO1 activity by analogs of DC-I028.

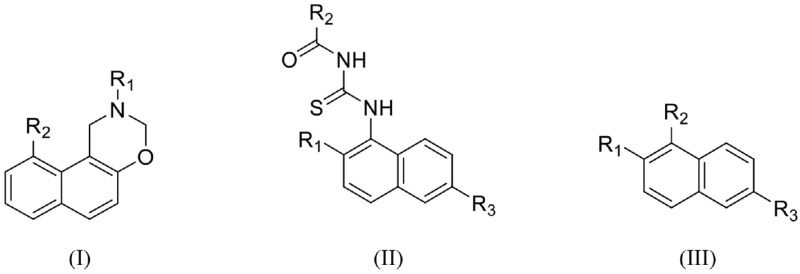							
**Cpd No**.^*a^	**Scaffold**	**R**_1_	**R**_2_	**R**_3_	**Inhibition ratio % (20 μM)**	**Enzymatic IC_50_ (μM)**	**Cellular IC_50_ (μM)**
DC-I028	(I)	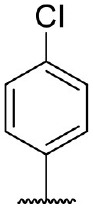	H	–^*b^	57.62	21.61	89.11
DC-I02801		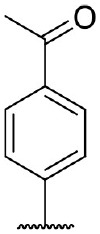	H	–	17.81	NT^*c^	NT
DC-I02802		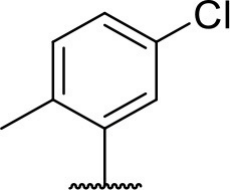	H	–	42.16	44.16	56.01
DC-I02803		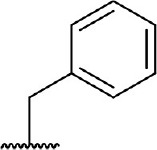	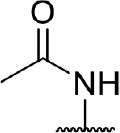	–	−11.17	NT	NT
DC-I02804		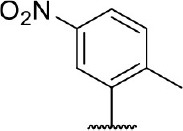	H	–	39.11	27.75	98.42
DC-I02805	(II)	OH	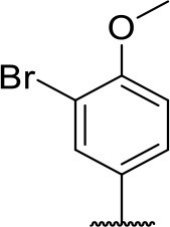	H	54.69	13.74	No activity
DC-I02806		OH	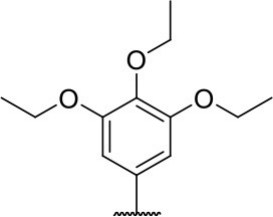	H	75.31	18.70	18.21
DC-I02807		OH	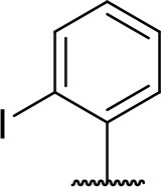	H	59.59	24.97	NT
DC-I02808		OH	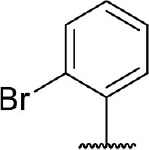	H	61.28	17.93	46.35
DC-I02809		H	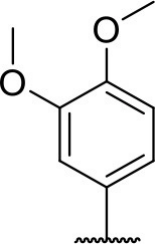	OH	17.55	NT	NT
DC-I02810		H	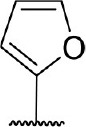	OH	15.60	NT	NT
DC-I02811	(III)	OH	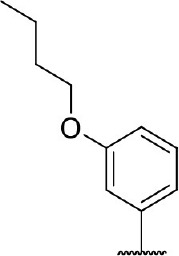	H	8.19	NT	NT
DC-I02812		OH	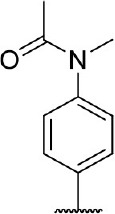	H	4.69	NT	NT
DC-I02813		OH	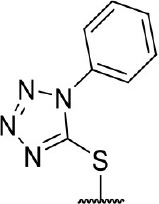	H	15.60	NT	NT

*a *Cpd No., compound number*.

*b *“–” represents not applicable*.

*c *“NT” represents not tested*.

## Conclusion

IDO1 plays a significant role in the proliferation and differentiation of T cells through the catabolism of tryptophan. Overexpression of IDO1 acts as the guard of T-cell attacking for many cancer types. In this study, structure-based virtual screening was used to discover new small-molecule inhibitors of IDO1 from compound library. A total of 104 compounds were selected and evaluated by an IDO1 enzymatic assay, in which DC-I028 showed the potent inhibitory activity against IDO1 with an IC_50_ of 21.61 μM on molecular level and 89.11 μM on Hela cellular level. Then, 13 analogs of DC-I028 obtained from hit expansion were evaluated. These analogs also demonstrated inhibitory activity against IDO1, among which DC-I02806 shows the highest inhibitory activity with IC_50_ about 18 μM for both enzymatic assay and cell-based assay. Overall, in this study we discovered a series of IDO1 inhibitors with a novel scaffold using the structure-guided drug design approach, which provides a useful starting point and information for the further development of cancer therapy and the related chemical biology studies.

## Author contributions

GZ, JX, XL, MZ, and SY: designed research; GZ, JX, DL, and JZ: performed research; YW, LW, YY, and DL: analyzed data; GZ, JX, and MZ: wrote the paper.

### Conflict of interest statement

The authors declare that the research was conducted in the absence of any commercial or financial relationships that could be construed as a potential conflict of interest.

## Abbreviations

IDO1: indoleamine 2,3-dioxygenase 1
IDO2: indoleamine 2,3-dioxygenase 2
TDO: tryptophan 2,3-dioxygenase
NMDA: N-methyl-D-aspartate
NAD: nicotinamide adenine dinucleotide
TAAs: tumor associated antigens
1-MT: 1-methyl-tryptophan
PAINS: pan-assay interference compounds
SP: standard precision
AUC: area under the receiver operating characteristic curve
ROC: receiver operation characteristic
RIE: robust initial enhancement
EF: enrichment factors
