# Supplementary material for: Discovery of Novel Inhibitors of Indoleamine 2,3-Dioxygenase 1 Through Structure-Based Virtual Screening
Source: Front Pharmacol. 2018 Mar 29;9:277. doi: 10.3389/fphar.2018.00277 (PMC5884943; doi:10.3389/fphar.2018.00277)
Supplement: Supplementary file 1 [file Table1.docx]

**SUPPLEMENTARY MATERIAL**

Discovery of Novel Inhibitors of Indoleamine 2, 3-Dioxygenase 1 through Structure-Based Virtual Screening

**Guoqing Zhang^1,2, †^, Jing Xing^2,3, †^, Yulan Wang^2,3^, Yan Ye^2,3^, Jihui Zhao^2,3^, Kaixian Chen^2^, Hualiang Jiang^2^, Mingyue Zheng^2,*^, Shiying Yan^1,*^**

^1^School of the Physical Sciences, Qingdao University, Qingdao 266071, China

^2^Drug Discovery and Design Center, State Key Laboratory of Drug Research, Shanghai Institute of Materia Medica, Chinese Academy of Sciences, 555 Zu Chongzhi Road, Shanghai 201203, China.

^3^ Department of Pharmacy, University of Chinese Academy of Sciences, 19A Yuquan Road, Beijing 100049, China.

^†^Contributed equally to this work.

^*^Corresponding Authors:

Mingyue Zheng

E-mail: myzheng@simm.ac.cn (M.Z.)

Shiying Yan

E-mail: ysy5954418@163.com (S.Y.)

**Contents**

1. Table S1. Published IDO1 inhibitors collected for virtual screening model validation.

2. Table S2. Docking scores and inhibitory activities against IDO1 of the candidate compounds from virtual screening.

**Table S1. Published IDO1 inhibitors collected for virtual screening model validation.**

| **Name** | **Structure** | **Reference** |
| --- | --- | --- |
| 680C91 |  | (Dolusic and Frederick, 2013) |
| LM10 |  | (Dolusic and Frederick, 2013) |
| 1MT |  | (Dolusic and Frederick, 2013) |
| MTH-Trp |  | (Dolusic and Frederick, 2013) |
| Brassinin |  | (Dolusic and Frederick, 2013) |
| PIM |  | (Dolusic and Frederick, 2013) |
| Annulin B |  | (Dolusic and Frederick, 2013) |
| Annulin C |  | (Dolusic and Frederick, 2013) |
| Indol-2-ylethanones |  | (Dolusic and Frederick, 2013) |
| Exiguamine A |  | (Dolusic and Frederick, 2013) |
| Camalexin |  | (Moldrup et al., 2013) |
| Brassilexin |  | (Sono, 1989) |
| MTH-benz[e]trptoline  Toho-1 |  | (Yamahira et al., 2014) |
| 1a |  | (Cheng et al., 2014) |
| 2o |  | (Cheng et al., 2014) |
| Newlink 1153 |  | (Dolusic and Frederick, 2013) |
| Newlink 1242 |  | (Dolusic and Frederick, 2013) |
| Newlink 1192 |  | (Dolusic and Frederick, 2013) |
| Newlink 1248 |  | (Dolusic and Frederick, 2013) |
| Newlink 1247 |  | (Dolusic and Frederick, 2013) |
| Newlink 1260 |  | (Dolusic and Frederick, 2013) |
| Newlink 1305 |  | (Dolusic and Frederick, 2013) |
| Newlink 1368 |  | (Dolusic and Frederick, 2013) |
| Newlink 1333 |  | (Dolusic and Frederick, 2013) |
| Newlink 1304 |  | (Dolusic and Frederick, 2013) |
| Newlink 1356 |  | (Dolusic and Frederick, 2013) |
| Benzothiazole |  | (Dolusic and Frederick, 2013) |
| Phenyl-thiazole |  | (Dolusic and Frederick, 2013) |
| Phenyl-triazole |  | (Dolusic and Frederick, 2013) |
| 3-HAA |  | (Dolusic and Frederick, 2013) |
| Lankenau 12 |  | (Dolusic and Frederick, 2013) |
| Lankenau 16 |  | (Dolusic and Frederick, 2013) |
| Lankenau 20 |  | (Dolusic and Frederick, 2013) |
| Lankenau 15 |  | (Dolusic and Frederick, 2013) |
| Adociaquinone B |  | (Dolusic and Frederick, 2013) |
| Xestoquinone |  | (Dolusic and Frederick, 2013) |
| Adociaquinone A |  | (Dolusic and Frederick, 2013) |
| 1,4-Naphthoquinone |  | (Dolusic and Frederick, 2013) |
| Juglone |  | (Dolusic and Frederick, 2013) |
| Dichlone |  | (Dolusic and Frederick, 2013) |
| Epacadostat |  | (Yue et al., 2017) |
| Indoximod |  | (Dolusic and Frederick, 2013) |
| AMG-1 |  | (Meininger et al., 2011) |
| Caulerpin |  | (Vottero et al., 2006) |
| 5m |  | (Dolusic and Frederick, 2013) |
| Ideal 48 |  | (Dolusic and Frederick, 2013) |
| Ideal 77 |  | (Dolusic and Frederick, 2013) |
| Ideal 76 |  | (Dolusic and Frederick, 2013) |
| Ideal 53 |  | (Dolusic and Frederick, 2013) |
| Exiguamine B |  | (Dolusic and Frederick, 2013) |
| Incyte c3 |  | (Dolusic and Frederick, 2013) |
| Incyte c4 |  | (Dolusic and Frederick, 2013) |
| Lankenau 28 |  | (Dolusic and Frederick, 2013) |
| Newlink 00568 |  | (Dolusic and Frederick, 2013)  (Mautino et al., 2007) |
| Dehydro-lapachone |  | (Peng et al., 2016) |
| B-lapachone |  | (Flick et al., 2013) |
| B-carboline |  | (Muller et al., 2005) |
| Tsitsikammamine A |  | (Dolusic and Frederick, 2013)  (Dolušić et al., 2013) |
| EGCG |  | (Chung et al., 2014) |
| Tryptanthrins |  | (Yang et al., 2013) |
| LICR 45 |  | (Dolusic and Frederick, 2013) |
| 2a |  | (Cheng et al., 2014) |
| 2d |  | (Cheng et al., 2014) |
| 2c |  | (Cheng et al., 2014) |
| 2f |  | (Cheng et al., 2014) |
| 2t |  | (Cheng et al., 2014) |
| 2i |  | (Cheng et al., 2014) |
| 3a |  | (Cheng et al., 2014) |
| 3b |  | (Cheng et al., 2014) |
| 3d |  | (Cheng et al., 2014) |
| 3c |  | (Cheng et al., 2014) |
| 5l |  | (Dolusic and Frederick, 2013) |
| 5k |  | (Dolusic and Frederick, 2013) |
| 8 |  | (Dolusic and Frederick, 2013) |
| 5a |  | (Dolusic and Frederick, 2013) |
| 5e |  | (Dolusic and Frederick, 2013) |
| Incyte c15 |  | (Dolusic and Frederick, 2013) |
| Incyte c13 |  | (Dolusic and Frederick, 2013) |
| LICR 41 |  | (Dolusic and Frederick, 2013) |
| LICR 36 |  | (Dolusic and Frederick, 2013) |
| LICR 46 |  | (Dolusic and Frederick, 2013) |
| LICR 47 |  | (Dolusic and Frederick, 2013) |
| LICR 40 |  | (Dolusic and Frederick, 2013) |
| NLG919 |  | (Peng et al., 2016) |
| 53 |  | (Rohrig et al., 2010) |
| Canesten |  | (Bakmiwewa et al., 2012) |
| ECONAZOLE |  | (Bakmiwewa et al., 2012) |
| Sildenafil |  | (Bakmiwewa et al., 2012) |
| Nifedipine |  | (Bakmiwewa et al., 2012) |
| NRB04258 |  | (Rohrig et al., 2014) |

**Table S2. Docking scores and inhibitory activities against IDO1 of the candidate compounds from virtual screening.**

| **CPD No.** | **Specs ID-number** | **Docking Score** | **Avg_Inh (%)**  **@ 50uM** | **Background (%)** |
| --- | --- | --- | --- | --- |
| DCI-001 | AO-476/41541790 | -7.67 | -9.13 | 42.64 |
| DCI-002 | AO-548/43301457 | -8.05 | 81.62 | 44.08 |
| DCI-003 | AK-918/41945294 | -8.00 | 35.38 | 51.94 |
| DCI-004 | AG-401/11435623 | -8.45 | 49.29 | 38.90 |
| DCI-005 | AG-670/36581045 | -8.10 | 11.86 | 36.51 |
| DCI-006 | AK-918/42813920 | -8.34 | 2.62 | 34.97 |
| DCI-007 | AM-807/14956047 | -7.78 | -0.85 | 44.80 |
| DCI-008 | AM-807/42003378 | -8.21 | 22.90 | 42.25 |
| DCI-009 | AI-204/31729036 | -7.96 | 12.24 | 52.98 |
| DCI-010 | AI-204/31721056 | -7.68 | 18.26 | 46.13 |
| DCI-011 | AT-051/43410109 | -7.90 | 2.22 | 51.96 |
| DCI-012 | AP-853/41543954 | -7.69 | 42.86 | 56.72 |
| DCI-013 | AP-853/43405437 | -7.83 | 4.76 | 38.66 |
| DCI-014 | AK-778/43206403 | -7.81 | 28.34 | 41.98 |
| DCI-015 | AK-968/15253562 | -7.75 | 20.11 | 40.12 |
| DCI-016 | AQ-390/43364090 | -7.97 | 5.08 | 38.92 |
| DCI-017 | AO-476/40923368 | -7.84 | 37.65 | 48.33 |
| DCI-018 | AN-465/43411374 | -8.49 | -2.06 | 34.42 |
| DCI-019 | AN-465/43421684 | -7.94 | -8.21 | 33.33 |
| DCI-020 | AK-918/42814086 | -8.35 | 10.24 | 35.53 |
| DCI-021 | AP-853/42876177 | -8.15 | -2.20 | 37.17 |
| DCI-022 | AO-080/43441703 | -7.82 | 34.49 | 44.32 |
| DCI-023 | AN-829/40406004 | -7.94 | -2.26 | 53.84 |
| DCI-024 | AK-198/36493015 | -7.83 | 45.39 | 56.24 |
| DCI-025 | AE-641/02401020 | -8.44 | 9.07 | 66.83 |
| DCI-026 | AS-871/11285277 | -8.07 | 98.78 | 66.01 |
| DCI-027 | AS-871/43475393 | -7.94 | 43.67 | 56.25 |
| DCI-028 | AP-906/14617008 | -7.89 | 82.33 | 55.25 |
| DCI-029 | AM-944/40948124 | -7.85 | -6.51 | 40.56 |
| DCI-030 | AE-562/12222611 | -7.93 | 11.57 | 45.02 |
| DCI-031 | AB-337/13036180 | -7.70 | 13.99 | 39.14 |
| DCI-032 | AG-690/11142277 | -7.87 | 63.02 | 33.85 |
| DCI-033 | AE-641/30102013 | -8.48 | 45.38 | 34.53 |
| DCI-034 | AO-365/43474714 | -7.73 | 5.04 | 42.24 |
| DCI-035 | AO-365/43474679 | -7.95 | 2.22 | 46.21 |
| DCI-036 | AP-970/40887165 | -8.55 | 76.32 | 74.58 |
| DCI-037 | AE-848/01427028 | -7.77 | 24.85 | 60.63 |
| DCI-038 | AK-029/34863025 | -8.36 | 6.52 | 60.07 |
| DCI-039 | AF-399/34136046 | -7.93 | -1.03 | 36.31 |
| DCI-040 | AK-968/41925721 | -8.00 | 40.66 | 31.86 |
| DCI-041 | AG-690/36281033 | -8.13 | 72.23 | 50.76 |
| DCI-042 | AP-836/41220136 | -7.76 | 59.00 | 48.79 |
| DCI-043 | AM-807/37225040 | -8.39 | 1.84 | 30.60 |
| DCI-044 | AM-944/40947553 | -8.16 | 44.52 | 40.43 |
| DCI-045 | AC-907/34131002 | -8.18 | 11.41 | 39.40 |
| DCI-046 | AP-263/40778176 | -7.74 | 63.18 | 44.38 |
| DCI-047 | AO-365/43403048 | -8.16 | -3.88 | 42.54 |
| DCI-048 | AO-833/41044554 | -7.78 | 7.58 | 71.51 |
| DCI-049 | AJ-292/14129470 | -7.75 | 1.65 | 31.42 |
| DCI-050 | AN-329/43450238 | -8.56 | 0.29 | 31.57 |
| DCI-051 | AQ-086/43457604 | -7.91 | 6.69 | 57.46 |
| DCI-052 | AN-465/42246307 | -8.45 | 3.32 | 31.34 |
| DCI-053 | AB-337/13036071 | -8.72 | 3.68 | 43.33 |
| DCI-054 | AI-204/31696055 | -7.93 | 4.51 | 48.55 |
| DCI-055 | AH-034/07273021 | -7.71 | 27.86 | 44.78 |
| DCI-056 | AH-262/31841014 | -7.82 | 55.55 | 43.42 |
| DCI-057 | AH-262/32338072 | -7.68 | 67.78 | 57.09 |
| DCI-058 | AK-968/13148469 | -7.81 | 4.69 | 37.75 |
| DCI-059 | AG-401/43167888 | -7.85 | 17.41 | 49.85 |
| DCI-060 | AS-871/43475343 | -7.68 | 79.74 | 59.25 |
| DCI-061 | AO-364/37357119 | -7.93 | 63.45 | 54.35 |
| DCI-062 | AJ-292/13095671 | -8.31 | 8.78 | 33.45 |
| DCI-063 | AK-968/40732719 | -8.00 | 2.41 | 54.16 |
| DCI-064 | AG-690/33370028 | -7.80 | 6.57 | 36.33 |
| DCI-065 | AN-698/40745382 | -7.92 | 26.49 | 68.09 |
| DCI-066 | AG-690/13780146 | -7.71 | 24.55 | 53.30 |
| DCI-067 | AG-690/11636137 | -7.88 | 28.06 | 37.16 |
| DCI-068 | AG-690/40697954 | -8.08 | 5.69 | 35.54 |
| DCI-069 | AN-943/40788806 | -8.38 | 79.20 | 42.53 |
| DCI-070 | AP-263/43302120 | -7.77 | 46.52 | 35.28 |
| DCI-071 | AF-399/42177233 | -8.92 | 22.41 | 39.05 |
| DCI-072 | AE-406/41056844 | -8.27 | 74.65 | 49.90 |
| DCI-073 | AP-381/41075787 | -8.34 | 64.05 | 54.63 |
| DCI-074 | AN-329/41609877 | -7.71 | 21.71 | 35.63 |
| DCI-075 | AK-918/41424401 | -7.83 | 79.98 | 55.42 |
| DCI-076 | AN-652/42917898 | -7.73 | 43.35 | 40.50 |
| DCI-077 | AI-204/33263035 | -7.68 | 51.64 | 57.47 |
| DCI-078 | AN-919/14790017 | -7.69 | -8.99 | 62.57 |
| DCI-079 | AM-807/43303361 | -7.84 | 85.60 | 60.62 |
| DCI-080 | AQ-405/42300372 | -7.77 | -3.23 | 48.38 |
| DCI-081 | AR-434/41599876 | -7.91 | -6.95 | 38.30 |
| DCI-082 | AO-548/43242483 | -7.71 | -2.26 | 35.32 |
| DCI-083 | AN-329/40602064 | -7.78 | 40.17 | 40.73 |
| DCI-084 | AN-329/40200631 | -7.89 | 35.60 | 64.61 |
| DCI-085 | AN-329/41328718 | -8.08 | -4.72 | 37.26 |
| DCI-086 | AG-670/36580056 | -8.30 | 77.96 | 50.53 |
| DCI-087 | AP-263/41670532 | -7.70 | -2.50 | 34.87 |
| DCI-088 | AG-690/11171046 | -8.23 | -0.32 | 43.06 |
| DCI-089 | AQ-086/43383878 | -7.78 | -4.37 | 35.22 |
| DCI-090 | AP-853/43368022 | -7.72 | 95.14 | 34.84 |
| DCI-091 | AN-465/42519155 | -7.71 | -6.47 | 41.00 |
| DCI-092 | AN-465/42519524 | -7.70 | 1.56 | 40.57 |
| DCI-093 | AN-329/43385611 | -7.72 | 2.13 | 35.52 |
| DCI-094 | AP-124/43383635 | -7.75 | -10.06 | 75.33 |
| DCI-095 | AF-399/41726129 | -7.87 | -1.55 | 43.56 |
| DCI-096 | AN-652/40090033 | -7.72 | 58.68 | 49.37 |
| DCI-097 | AQ-911/41962550 | -7.69 | -2.09 | 37.58 |
| DCI-098 | AM-807/42004484 | -8.10 | 4.10 | 35.87 |
| DCI-099 | AQ-750/41790303 | -8.10 | 27.63 | 49.90 |
| DCI-100 | AN-329/40439827 | -8.24 | 94.68 | 57.16 |
| DCI-101 | AE-848/07404040 | -7.83 | -0.96 | 35.16 |
| DCI-102 | AN-698/42116914 | -7.88 | 80.83 | 43.32 |
| DCI-103 | AJ-292/42151568 | -8.03 | 43.77 | 52.04 |
| DCI-104 | AK-968/12102663 | -8.31 | 25.72 | 41.51 |
| NLG919 |  |  | 100 | 32.90 |

*CPD, Compound; Avg, Average; Inh, Inhibition.

**References**

Bakmiwewa, S.M., Fatokun, A.A., Tran, A., Payne, R.J., Hunt, N.H., and Ball, H.J. (2012). Identification of selective inhibitors of indoleamine 2,3-dioxygenase 2. *Bioorganic & Medicinal Chemistry Letters* 22(24)**,** 7641-7646. doi: <https://doi.org/10.1016/j.bmcl.2012.10.010>.

Cheng, M.F., Hung, M.S., Song, J.S., Lin, S.Y., Liao, F.Y., Wu, M.H., et al. (2014). Discovery and structure-activity relationships of phenyl benzenesulfonylhydrazides as novel indoleamine 2,3-dioxygenase inhibitors. *Bioorg Med Chem Lett* 24(15)**,** 3403-3406. doi: 10.1016/j.bmcl.2014.05.084.

Chung, J.E., Tan, S., Gao, S.J., Yongvongsoontorn, N., Kim, S.H., Lee, J.H., et al. (2014). Self-assembled micellar nanocomplexes comprising green tea catechin derivatives and protein drugs for cancer therapy. *Nat Nanotechnol* 9(11)**,** 907-912. doi: 10.1038/nnano.2014.208.

Dolusic, E., and Frederick, R. (2013). Indoleamine 2,3-dioxygenase inhibitors: a patent review (2008 - 2012). *Expert Opin Ther Pat* 23(10)**,** 1367-1381. doi: 10.1517/13543776.2013.827662.

Dolušić, E., Larrieu, P., Meinguet, C., Colette, D., Rives, A., Blanc, S., et al. (2013). Indoleamine 2,3-dioxygenase inhibitory activity of derivatives of marine alkaloid tsitsikammamine A. *Bioorganic & Medicinal Chemistry Letters* 23(1)**,** 47-54. doi: <https://doi.org/10.1016/j.bmcl.2012.11.036>.

Flick, H.E., Lalonde, J.M., Malachowski, W.P., and Muller, A.J. (2013). The Tumor-Selective Cytotoxic Agent beta-Lapachone is a Potent Inhibitor of IDO1. *Int J Tryptophan Res* 6**,** 35-45. doi: 10.4137/ijtr.s12094.

Mautino, M., Jaipuri, F., Marcinowicz-Flick, A., Kesharwani, T., Waldo, J., and Collier, S.J. (2007). *IDO Inhibitors*. America patent application US20110053941.

Meininger, D., Zalameda, L., Liu, Y., Stepan, L.P., Borges, L., McCarter, J.D., et al. (2011). Purification and kinetic characterization of human indoleamine 2,3-dioxygenases 1 and 2 (IDO1 and IDO2) and discovery of selective IDO1 inhibitors. *Biochim Biophys Acta* 1814(12)**,** 1947-1954. doi: 10.1016/j.bbapap.2011.07.023.

Moldrup, M.E., Geu-Flores, F., and Halkier, B.A. (2013). Assigning gene function in biosynthetic pathways: camalexin and beyond. *Plant Cell* 25(2)**,** 360-367. doi: 10.1105/tpc.112.104745.

Muller, A.J., Malachowski, W.P., and Prendergast, G.C. (2005). Indoleamine 2,3-dioxygenase in cancer: targeting pathological immune tolerance with small-molecule inhibitors. *Expert Opin Ther Targets* 9(4)**,** 831-849. doi: 10.1517/14728222.9.4.831.

Peng, Y.H., Ueng, S.H., Tseng, C.T., Hung, M.S., Song, J.S., Wu, J.S., et al. (2016). Important Hydrogen Bond Networks in Indoleamine 2,3-Dioxygenase 1 (IDO1) Inhibitor Design Revealed by Crystal Structures of Imidazoleisoindole Derivatives with IDO1. *J Med Chem* 59(1)**,** 282-293. doi: 10.1021/acs.jmedchem.5b01390.

Rohrig, U.F., Awad, L., Grosdidier, A., Larrieu, P., Stroobant, V., Colau, D., et al. (2010). Rational design of indoleamine 2,3-dioxygenase inhibitors. *J Med Chem* 53(3)**,** 1172-1189. doi: 10.1021/jm9014718.

Rohrig, U.F., Majjigapu, S.R., Chambon, M., Bron, S., Pilotte, L., Colau, D., et al. (2014). Detailed analysis and follow-up studies of a high-throughput screening for indoleamine 2,3-dioxygenase 1 (IDO1) inhibitors. *Eur J Med Chem* 84**,** 284-301. doi: 10.1016/j.ejmech.2014.06.078.

Sono, M. (1989). Enzyme kinetic and spectroscopic studies of inhibitor and effector interactions with indoleamine 2,3-dioxygenase. 2. Evidence for the existence of another binding site in the enzyme for indole derivative effectors. *Biochemistry* 28(13)**,** 5400-5407.

Vottero, E., Balgi, A., Woods, K., Tugendreich, S., Melese, T., Andersen, R.J., et al. (2006). Inhibitors of human indoleamine 2,3-dioxygenase identified with a target-based screen in yeast. *Biotechnol J* 1(3)**,** 282-288. doi: 10.1002/biot.200600001.

Yamahira, A., Narita, M., Iwabuchi, M., Uchiyama, T., Iwaya, S., Ohiwa, R., et al. (2014). Activation of the leukemia plasmacytoid dendritic cell line PMDC05 by Toho-1, a novel IDO inhibitor. *Anticancer Res* 34(8)**,** 4021-4028.

Yang, S., Li, X., Hu, F., Li, Y., Yang, Y., Yan, J., et al. (2013). Discovery of tryptanthrin derivatives as potent inhibitors of indoleamine 2,3-dioxygenase with therapeutic activity in Lewis lung cancer (LLC) tumor-bearing mice. *J Med Chem* 56(21)**,** 8321-8331. doi: 10.1021/jm401195n.

Yue, E.W., Sparks, R., Polam, P., Modi, D., Douty, B., Wayland, B., et al. (2017). INCB24360 (Epacadostat), a Highly Potent and Selective Indoleamine-2,3-dioxygenase 1 (IDO1) Inhibitor for Immuno-oncology. *ACS Med Chem Lett* 8(5)**,** 486-491. doi: 10.1021/acsmedchemlett.6b00391.
